# Harnessing PTEN’s Growth Potential in Neuronal Development and Disease

**DOI:** 10.1177/2633105520959056

**Published:** 2020-09-13

**Authors:** Joachim Fuchs, Britta J. Eickholt, George Leondaritis

**Affiliations:** 1 Institute of Molecular Biology and Biochemistry, Charité – Universitätsmedizin Berlin, Germany; 2 Department of Medicine, Laboratory of Pharmacology, University of Ioannina, Ioannina, Epirus, Greece

**Keywords:** PTEN, axonal branches, development, Plasticity related genes, PRG2, lipid phosphate phosphatase related, LPPR3, axonal growth, Phosphatidylinositol 3,4,5-trisphosphate

## Abstract

PTEN is a powerful regulator of neuronal growth. It globally suppresses axon extension and branching during both nervous system development and regeneration, by antagonizing growth-promoting PI3K/PI(3,4,5)P_3_ signaling. We recently identified that the transmembrane protein PRG2/LPPR3 functions as a modulator of PTEN function during axon morphogenesis. Our work demonstrates that through inhibition of PTEN activity, PRG2 stabilizes membrane PI(3,4,5)P_3_. In turn, PRG2 deficiency attenuates the formation of branches in a PTEN-dependent manner, albeit without affecting the overall growth capacity of extending axons. Thus, PRG2 is poised to temporally and locally relieve growth suppression mediated by PTEN in neurons and, in effect, to redirect growth specifically to axonal branches. In this commentary, we discuss potential implications and unresolved questions regarding the regulation of axonal PTEN in neurons. Given their widespread implication during neuronal development and regeneration, identification of mechanisms that confer spatiotemporal control of PTEN may unveil new approaches to reprogram PI3K signaling in neurodevelopmental disorders and regeneration research.

**Comment on:** Brosig A, Fuchs J, Ipek F, Kroon C, Schrötter S, Vadhvani M, Polyzou A, Ledderose J, van Diepen M, Holzhütter HG, Trimbuch T, Gimber N, Schmoranzer J, Lieberam I, Rosenmund C, Spahn C, Scheerer P, Szczepek M, Leondaritis G, Eickholt BJ. The Axonal Membrane Protein PRG2 Inhibits PTEN and Directs Growth to Branches. *Cell Rep*. 2019 Nov 12;29(7):2028-2040.e8. doi: 10.1016/j.celrep.2019.10.039. PubMed PMID: 31722215; PubMed Central PMCID: PMC6856728. https://pubmed.ncbi.nlm.nih.gov/31722215/

Cell growth is defined by upregulation of macromolecular synthesis and increases in both cell size and mass. As a fundamental cellular attribute, cell growth is under tight control by numerous signaling pathways and their associated transcriptional programs. An established growth-promoting signaling pathway in neurons is the phosphoinositide 3-kinase (PI3K) and phosphatase and tensin homolog deleted on chromosome 10 (PTEN) pathway. Many studies have highlighted the importance of PI3Ks and PTEN for neuronal axon elongation and branching during development as well as during regeneration following injury to the nervous system.^1,2^ PI3K synthesizes the second messenger phosphatidylinositol 3,4,5-trisphosphate (PI(3,4,5)P_3_), while PTEN hydrolyzes it.^1^ PI(3,4,5)P_3_ and the downstream Akt/mTORC1 growth-associated signaling pathway are indispensable for controlling multiple growth processes, while inactivation of PTEN is associated with cancers, overgrowth syndromes and autism spectrum disorders.^1,3^ Thus, it is apparent that tuning the balance between PI3K/PI(3,4,5)P_3_ and PTEN is crucial for adjusting growth associated signaling. In neurons, PTEN has been thought of as a brake for different aspects of axon and dendrite growth, including presynaptic terminals and dendritic spines.^1,2^ We are starting to appreciate the local aspects and the spatiotemporal control of PTEN in dendritic spines and their implication for neuronal physiology.^1,4^ In contrast, the spatiotemporal regulation of PTEN in the extending axon is much less explored even though PTEN inhibition has been highlighted for over a decade as a permissive factor for regeneration of injured axons.^1,2^

Under basal culture conditions, PI(3,4,5)P_3_ is highly abundant in developing axons despite high levels of PTEN (Figure 1). However, axonal PI(3,4,5)P_3_ levels do not remain high throughout differentiation in culture; a recent study showed that significant downregulation of axonal PI(3,4,5)P_3_ takes place as CNS neurons mature to form synapses and this correlates with loss of regenerative ability in mature neurons.^5^ PTEN is generally considered to be constitutively active which raises the question of how neurons can intrinsically sustain PI(3,4,5)P_3_-rich axonal membrane domains in the presence of high levels of active axonal PTEN during these early stages in axonal growth.

**Figure 1.**
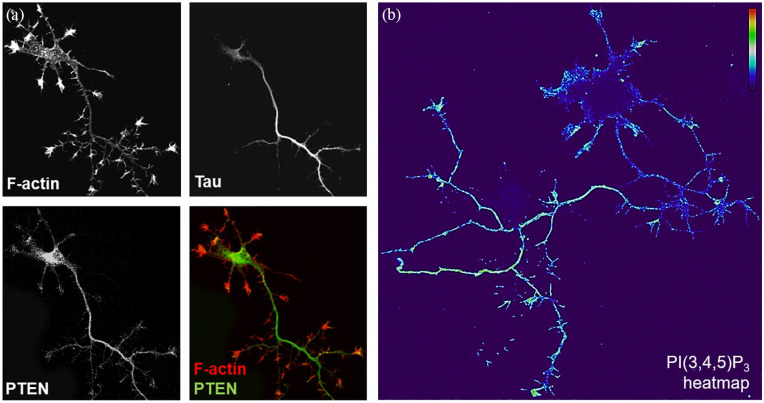
PTEN and PI(3,4,5)P_3_ are highly abundant in developing axonal shafts. (a) DIV5 primary hippocampal neuron fixed and immunostained with anti-Tau1, anti-PTEN antibodies and phalloidin. Immunolabeling reveals a prominent enrichment of PTEN in the Tau1-positive axon and major nascent branches. (b) A DIV5 hippocampal neuron stained with anti-PI(3,4,5)P_3_ antibodies, using a lipid-specific staining protocol,^5^ reveals high PI(3,4,5)P_3_ levels in the growing primary axon, filopodial protrusions, nascent branches and growth cones.

## PRG2 inhibits PTEN and directs growth to branches

While studying PTEN-interacting neuronal proteins, we recently uncovered a neuronal membrane protein complex involving an axonal transmembrane protein, called PRG2, (Plasticity Related Gene 2; also known as LPPR3, Lipid phosphate phosphatase Related 3). We show that PRG2 exhibits a striking localization pattern of sub-micrometer size clusters along the axonal shaft membrane and its expression correlates with phases of axonal growth and branching in vitro and in vivo. PRG2 loss-of function and gain-of-function causes decreases and increases in axonal filopodia and branches, respectively, with virtually no growth defects in soma and dendrites.^6^ Our work further identified that PRG2 is able to bind to PTEN and inhibit its PI(3,4,5)P_3_-phosphatase activity. Importantly, all alterations in axonal filopodia and branches caused by PRG2 knockout or overexpression were reversed by correcting PI(3,4,5)P_3_ levels using genetic or pharmacological manipulation of PI3K and PTEN activities.^6^ In conclusion, our work identifies that PRG2 may function as a developmentally interposed switch directing neuronal growth specifically toward axonal branches via control of PTEN activity.

**Figure 2.**
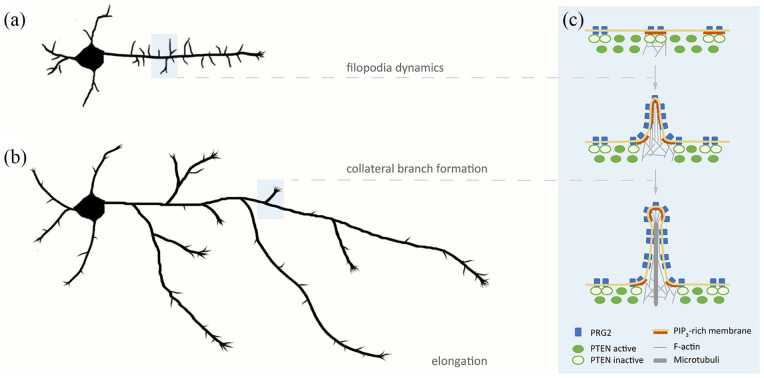
Patterns of axonal growth. (a) Newly formed axons experience a rapid growth phase from the distal growth cone and are decorated by numerous filopodial protrusions that are highly dynamic. (b) In later stages, ensuing axonal growth from distal growth cones is accompanied by formation of dynamic collateral branches. (c) Enlarged view of the axonal plasma membrane (PM) where PRG2 localized in nanodomains and clusters, binds and locally inhibits PTEN in the axon. This supports the stabilization of PI(3,4,5)P_3_-rich axonal PM domains which can function as signals for F-actin polymerization and initial filopodia formation that subsequently can form nascent branches by invasion and stabilization of microtubules.

Axonal growth can manifest as elongation and/or branching (Figure 2). Both aspects coordinate with the overall neuronal growth capacity of neurons, yet, they can be separated developmentally, operationally and even mechanistically.^7,8^ Axonal collateral branching is fundamental for the assembly of interconnected neuronal networks in the adult brain and is found deregulated in many neurodevelopmental and psychiatric diseases in humans.^7^ It is established that PI3K/PTEN and PI(3,4,5)P_3_ are essential for the proper reorganization of the plasma membrane actin cytoskeleton in order to support formation of filopodial protrusions along the axonal shaft and at the leading edge of axonal growth cones.^1,7^ Axonal shaft filopodia generally are considered precursors of nascent branches, which mature by subsequent invasion and stabilization of microtubules.^9,10^

Our biochemical and cell biology experiments with PRG2 and PTEN suggest a model, where PRG2 stabilizes PI(3,4,5)P_3_-rich membrane domains and F-actin-rich membrane protrusions along the axonal shaft by sequestering and inhibiting PTEN (Figure 2c). In this mechanism, PRG2 operates as a permissive factor for formation of filopodia and branches. It also suggests the existence of additional interactions of PRG2 with PI(3,4,5)P_3_, other phosphoinositides and/or F-actin regulators. A number of observations point to a unique function of PRG2 for generating filopodia, as opposed to other F-actin structures like lamellipodia that propel axonal branching and growth, and are also controlled by PI3K/PTEN/PI(3,4,5)P_3_ signaling.^7^ On the one hand, PRG2, when overexpressed in cells, is enriched in filopodial-type membrane protrusions as opposed to lamellipodial membranes (Leondaritis, Eickholt, unpublished). On the other hand, deletion of PRG2 in neurons reduces the formation of axonal filopodia-originating branches, leaving axonal lamellipodia-originating branches unaffected (Fuchs, Eickholt, unpublished). These observations suggest a remarkable specificity of PRG2 to control of PTEN/PI(3,4,5)P_3_-dependent F-actin structures.

Axon growth can be controlled by signaling events in the soma, by axonal transport mechanisms, and by local regulation of axonal cytoskeleton. Establishing propulsive activity in axonal growth cones and branches are particular important in executing the neuron’s growth ability and involves tools that operate specifically in the different membrane domains of axons. Indeed, localized ‘turnover’ of PI(3,4,5)P_3_ can influence the formation of F-actin patches, lamellipodia and filopodia along the axonal shaft membrane.^10^ In the axon, PI(3,4,5)P_3_ is found in accumulations along the axon membrane with occasional strong puncta seen at the base (or in vicinity) of emerging filopodia or membrane protrusions (Figure 1b; Nieuwenhuis et al^5^). Although the regulation of PI(3,4,5)P_3_ at the vicinity of the growth cone during attraction or repulsion by extracellular cues or during differentiation is well described,^5,10^ we lack understanding of how PI(3,4,5)P_3_ (or other phosphoinositide) microdomains operate along the axon shaft. Given that inhibition of PTEN in the axonal compartment can counteract its growth-restricting function,^11^ we hypothesize that a neuron-autonomous local regulation of PI(3,4,5)P_3_ by PI3K and PTEN activities along the axon is instrumental in fine-tuning when and where an axon will branch.

## Global versus local regulation of axonal PTEN and PI(3,4,5)P_3_ - unresolved questions and implications

Our model highlights the idea that PRG2 can function in promoting neuronal growth toward axonal branches, and we surmise that local inhibition of axonal PTEN during development may be the driving force for such spatially confined growth responses. Injury to the CNS such as spinal cord injury (SCI) causes a failure to re-establish neuronal connectivity, which is partially due to the fact that axons exhibit a lack of an intrinsic regenerative growth response. It is now established that global inhibition of PTEN by chemical and genetic manipulation boosts axonal growth responses following SCI.^1,2^ Similarly, activation of PI3K/PI(3,4,5)P_3_ has been shown to induce axonal growth after injury in different neuronal populations.^2,5^ Thus, in the context of axon regeneration one may consider to harness PTEN’s multifaceted growth controlling potential to distinguish effects that specifically target elongation from those inducing collateral branches. In this respect, treatments locally inducing branching to facilitate the formation of compensatory relay networks,^12^ and treatments suppressing branch formation to ensure growth potential is efficiently directed toward elongation – involving localized rather than global regulatory mechanisms targeting PTEN function – could be strategies to regain connectivity and function in the context of SCI.

PRG2 belongs to a developmentally regulated, neuron-enriched, protein family that share common phylogeny and structure with type II lipid phosphate phosphatases that hydrolyze the bioactive lipid lyso-phosphatidic acid (LPA). Despite being inactive as phosphatases, PRG proteins still appear to function in a LPA-dependent manner, for example as LPA effectors or sensors, or even as LPA scavengers to control synaptic activity, axonal growth and growth cone navigation.^13,14^ Interestingly, another PRG protein, namely PRG3 (or LPPR1), was recently shown to promote axonal regeneration in a LPA-dependent manner.^13^ Given the established propensity of these proteins to form heteromeric complexes,^6,15^ it is likely that the PRG2-PTEN interaction may also operate in the form of PRG multimers. This mechanism may well provide an additional effector pathway for extracellular bioactive lipids like LPA for tuning neuronal cell morphology and growth during development and after trauma.
